# A rat decellularized small bowel scaffold that preserves villus-crypt architecture for intestinal regeneration

**DOI:** 10.1016/j.biomaterials.2012.01.012

**Published:** 2012-04

**Authors:** Giorgia Totonelli, Panagiotis Maghsoudlou, Massimo Garriboli, Johannes Riegler, Giuseppe Orlando, Alan J. Burns, Neil J. Sebire, Virpi V. Smith, Jonathan M. Fishman, Marco Ghionzoli, Mark Turmaine, Martin A. Birchall, Anthony Atala, Shay Soker, Mark F. Lythgoe, Alexander Seifalian, Agostino Pierro, Simon Eaton, Paolo De Coppi

**Affiliations:** a Surgery Unit, Institute of Child Health and Great Ormond Street Hospital, University College London, 30 Guilford Street, London WC1N 1EH, UK; b Centre for Advanced Biomedical Imaging, Department of Medicine and Institute of Child Health, University College London, London WC1N 1EH, UK; c Wake Forest Institute for Regenerative Medicine, Wake Forest University School of Medicine, Winston-Salem, NC 27157, USA; d Neural Development Unit, UCL Institute of Child Health, University College London, London WC1N 1EH, UK; e Department of Histopathology, Institute of Child Health and Great Ormond Street Hospital, University College London, London WC1N 1EH, UK; f Division of Bioscience, University College London, London WC1N 1EH, UK; g UCL Ear Institute, London WC1X 8EE, UK; h Research Department of General Surgery, Royal Free Hospital, University College London, London NW3 2PF, UK

**Keywords:** Intestinal failure, Decellularization, Natural acellular matrix, Tissue engineering, Regenerative medicine, Gut transplantation

## Abstract

Management of intestinal failure remains a clinical challenge and total parenteral nutrition, intestinal elongation and/or transplantation are partial solutions. In this study, using a detergent-enzymatic treatment (DET), we optimize in rats a new protocol that creates a natural intestinal scaffold, as a base for developing functional intestinal tissue. After 1 cycle of DET, histological examination and SEM and TEM analyses showed removal of cellular elements with preservation of the native architecture and connective tissue components. Maintenance of biomechanical, adhesion and angiogenic properties were also demonstrated strengthen the idea that matrices obtained using DET may represent a valid support for intestinal regeneration.

## 1 Introduction

Intestinal failure (IF), arising from anatomical or functional loss of intestine, is a condition characterized by the inability of the intestine to carry out its secretory and absorptive functions, necessitating macronutrient, water and electrolyte supplementation, in the form of artificial feeding and parenteral nutrition (PN). IF reduces quality of life and has major PN-related co-morbidities such as liver disease, intravenous line sepsis and malnutrition. This leads to a survival rate of 86% in 1 year, which is reduced to 77% and 73% in 3 and 5 years respectively [1]. Non-transplant surgery such as the introduction of intestinal valves, reversed intestinal segments, and colon interposition has been used to wean patients off PN, but with debatable improvement [2]. In children, increase of the mucosal surface using intestinal lengthening techniques or tapering procedures has also been attempted but results are still controversial [3]. Small bowel transplantation (SBT) is an alternative, but limitations are related to the scarcity of donor organs, rejection, need for immunosuppression and survival rates of 60% in 5 years [4]. The need for age-matched donors in the pediatric population is an additional problem that affects the long-term success of SBT. Thus, the development of new treatment strategies for IF is an area requiring further investigation.

In 1999, the term “regenerative medicine” was first used to describe the use of natural human substances, such as genes, proteins, cells, and biomaterials to regenerate diseased or damaged human tissue [5,6] to restore normal function [7]. Within regenerative medicine, tissue engineering is concerned with the manufacturing of tissue by combining appropriate cells with a scaffold [8] that can be either synthetic such as poly-glycolic acid (PGA) [9,10], naturally-derived such as collagen or decellularized tissue [11,12]. Tissue engineering has been applied successfully in the clinic for the production of hollow structures that allow storage such as the bladder [9] and passage such as the urethra [10] and the trachea [13,14].

Previous attempts in intestinal tissue generation explored the use of intestinal epithelial organoid units (OUs) from neonatal rat small intestine seeded on PLGA [14]. When these constructs were implanted in rat and porcine animal models of short bowel syndrome there was partial restoration of gut function [15,16]. However, the experimental design does not allow clinical translation of this approach because of the inability to generate intestinal tissue from adult-derived OUs, practical difficulties in obtaining neonatal OUs for autologous transplantation and the fact that none of the previous studies have been able to generate intestine of equal or greater length to the unit used to generate the OUs. Major limitations of this approach are the high number of cells necessary for engineering functional tissue and the lack of a matrix that will mimic the original intestine, increase cell growth and form the intestinal stem cell niche.

Decellularization is an attractive technique for intestinal scaffold generation because of the possibility of retaining the architecture of the native tissue including the vasculature and biofactors that are present in the extracellular matrix (ECM) and that are needed for cell proliferation [17]. We have previously shown that muscle tissue can be successfully decellularized [18] and used for repair of a surgically created defect of the abdominal wall [19,20] and the diaphragm [21]. We and others have also shown that the trachea can be easily decellularized with the same process in pigs and humans, maintaining suitable characteristics for regeneration that have led to its clinical application [11–13]. Recently, various groups have successfully decellularized an entire heart [22], lung [23], liver [24,25], kidney [26] and pancreas [27]. Even if the engineering of such complex modular organs remains distant for any clinical application, rapid advancement in understanding ECM structure of these organs in physiological and pathological situations may help to develop strategies for their repair. Relatively simpler, but definitely more complex than the trachea, intestinal matrices could represent the basis for the development of an *in vitro* environment that more accurately resembles intestinal physiology [28,29]. This *in vitro* tissue-engineered intestine could be used as a low-cost and high-speed method to quickly explore toxic and non-functional compounds.

The aim of this work was to generate an acellular natural matrix from rat intestine able to maintain the intestinal architecture whilst removing all traces of donor-derived cells.

## 2 Methods

### 2.1 Organ harvest from rats

**Fig. 1:**
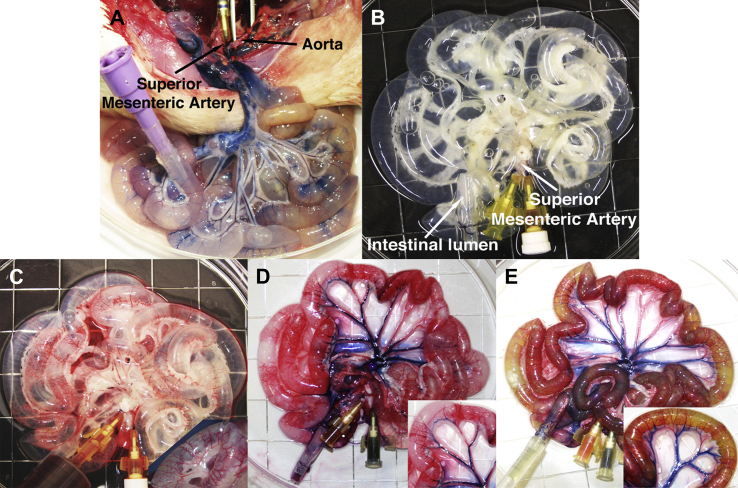
Decellularization of rat small intestine with detergent-enzymatic treatment. Macroscopic images prior (A) and following (B) one cycle of decellularization. Perfusion with Rosso Ponceau (C), Trypan blue (D) and Rhodamine green (E) dyes displays the patency and distribution of the arterial (C) and venous (D) capillary beds in contrast with the lumen (E) proving absence of leakage.

All surgical procedures and animal husbandry were carried out in accordance with UK Home Office guidelines under the Animals (Scientific Procedures) Act 1986 and the local ethics committee. Twenty-five adult Sprague–Dawley rats, weighing 320–350 g, were sacrificed by CO_2_ inhalation and cervical dislocation. Once sacrificed, a midline incision was made to completely expose the abdominal cavity. The superior mesenteric artery (SMA) was cannulated and flushed with phosphate buffered saline (PBS) to wash the vascular tree and prevent coagulation (Fig. 1A). The small intestine was dissected free and removed *en bloc* from pylorus to ileocecal valve and the intestinal lumen cannulated and washed with PBS containing 5% antibiotic-antimycotic solution (PBS/AA).

### 2.2 Detergent-enzymatic treatment (DET)

Both the intestinal lumen and the vascular tree were perfused with continuous fluid delivery using a Masterflex L/S variable speed roller pump at 0.6 ml/h. Each DET cycle was composed of deionized water (resistivity 18.2 MΩ/cm) at 4 °C for 24 h, 4% sodium deoxycholate (Sigma) at room temperature (RT) for 4 h, and 2000kU DNase-I (Sigma) in 1 M NaCl (Sigma) at RT for 3 h, as previously described [30]. After each treatment cycle the constructs were preserved at 4 °C, in PBS/AA.

### 2.3 Histological and immunostain analysis

Samples were fixed for 24 h in 10% neutral buffered formalin solution in PBS (pH 7.4) at RT. Subsequently they were washed in distilled water (dH2O), dehydrated in graded alcohol, embedded in paraffin and sectioned at 5 μm. Tissue slides were stained with Hematoxylin and Eosin (H&E) (Leica, Germany), Masson’s trichrome (MT), (Leica, Raymond A Lamb, BDH Chemicals Ltd), Picrosirius Red (PR) (Hopkin & Williams), Elastic Van Gieson (EVG) (VWR, Leica, Raymond A Lamb) and Alcian Blue (AB) (BDH Chemicals Ltd, Cellpath Ltd) stains. For the immunostaining analysis, the sections were incubated with mouse monoclonal antibody to major histocompatibility complex II (MHC-II) (Dako), Smooth Muscle Actin (Leica), Vimentin (Leica), Cytokeratin MNF 116 (Dako), Perls’ Prussian Blue (PB) (Merck, BDH Chemicals Ltd) and Cleaved Caspase 3 (Cell Signalling), according to standard immunostaining protocols using an avidin-biotin based detection system.

### 2.4 DNA quantification

To assess total DNA content within the native intestine and acellular matrices, specimens were disintegrated and homogenized in 1 mL lysis buffer, consisting of 50 mM Tris–HCl (pH 8), 50 mM EDTA, 1% SDS and 10 mM NaCl. Samples were digested with Proteinase K overnight, followed by phenol/chloroform extraction. The DNA was precipitated from the aqueous phase with 100% ethanol and washed with 70% ethanol. The pellet was then dissolved in ribonuclease-free water and stored at −20 °C. Subsequently, the extracts were characterized spectrophotometrically. Optical densities at 260 nm and 280 nm were used to estimate the purity and yield of nucleic acids, which were quantified on the basis of 280 nm absorbance.

### 2.5 Scanning electron microscopy (SEM)

Samples were fixed in 2% glutaraldehyde in 0.1 M phosphate buffer and left for 24 h at 3 °C. Following washing with 0.1 M phosphate buffer, they were cut into segments of approximately 1 cm length and cryoprotected in 25% sucrose, 10% glycerol in 0.05 M PBS (pH 7.4) for 2 h, then fast frozen in Nitrogen slush and fractured at approximately −160 °C. The samples were then placed back into the cryoprotectant at room temperature and allowed to thaw. After washing in 0.1 M phosphate buffer (pH 7.4), the material was fixed in 1% OsO_4_/0.1 M phosphate buffer (pH 7.3) at 3 °C for 1½ hours and washed again in 0.1 M phosphate buffer (pH 7.4). After rinsing with dH_2_O, specimens were dehydrated in a graded ethanol-water series to 100% ethanol, critical point dried using CO_2_ and finally mounted on aluminum stubs using sticky carbon taps. The fractured material was mounted to present fractured surfaces across the lumen wall to the beam. The complete samples were opened and mounted to show the lumen surface, then coated with a thin layer of Au/Pd (approximately 2 nm thick) using a Gatan ion beam coater. Images were recorded with a Jeol 7401 FEG scanning electron microscope.

### 2.6 Transmission electron microscopy (TEM)

Gut samples were cut into segments having a wall of approximately 1 cm in length. After washing in 0.1 M phosphate buffer (pH 7.4), they were fixed in 1% OsO_4_/0.1 M phosphate buffer (pH 7.3) at 3 °C for 1½ hours then washed in 0.1 M phosphate buffer (pH 7.4). Specimens were stained en bloc with 0.5% uranyl acetate in dH_2_0 at 3 °C for 30 min, rinsed with dH_2_O, dehydrated in a graded ethanol-water series and infiltrated with Agar 100 resin and then hardened. Sections measuring 1 μm were cut and stained with 1% toluidine blue in dH_2_O for light microscopy. A representative area was selected and sections were cut at 70–80 nm using a diamond knife on a Reichert ultra-cut E microtome. Sections were collected on 200-mesh copper, coated slot grid and stained with uranyl acetate and lead citrate. Images were recorded with a Joel 1010 transition electron microscope.

### 2.7 Collagen quantification

The collagen content of fresh and decellularized intestine was quantified using the SIRCOL collagen assay (Biocolor) according to the manufacturer’s instructions. Briefly, the samples were homogenized, and collagen was solubilized in 0.5 M acetic acid. Extracts were incubated with Sirius red dye, and absorbance was determined at 555 nm with a microplate reader (Tecan Infinity). Collagen concentrations from a standard curve were used to calculate the collagen content of the tissue.

### 2.8 Glycosaminoglycan quantification

The sulfated glycosaminoglycan (GAG) content of fresh and decellularized intestine was quantified using the Blyscan GAG Assay Kit (Biocolor, UK). In brief, 50 mg of minced wet tissue was weighed and placed in a micro-centrifuge tube containing 1 ml of Papain digestion buffer and incubated in a water bath at 65 °C for 18 h, with occasional tube removal and vortexing. Aliquots of each sample were mixed with 1,9-dimethyl-methylene blue dye and reagents from the GAG assay kit. The absorbance at 595 nm was measured using a microplate reader (Tecan Infinity) and compared to standards made from bovine tracheal chondroitin-4-sulfate to determine the absolute GAG content.

### 2.9 Biomechanical tests

The specimens were subjected to uniaxial tension until failure. This test records the tensile strength “*σ*” (Stress) versus strain “*ε*”; the highest point of the stress–strain curve is the Ultimate Tensile Strength (UTS). The ratio of stress to strain is the Young’s modulus, *E*, which is a measure of the stiffness of an elastic material. Mechanical tests were performed with the application of uniaxial tension in an Instron 5565 at room temperature (20 ± 1 °C). Specimens in the form of flat dumbbells with a 20 mm long working part were loaded at a constant tension rate of 100 mm/min. The thickness of the samples was measured using a digital electronic micrometer (RS components) at three places of the dumbbell and averaged. Stress–strain relationships, ultimate tensile strength (UTS), defined as maximum stress that a material could withstand until it breaks, and tensile modulus were obtained for samples and graphs plotted. Five samples were considered for each evaluated tissue.

### 2.10 Magnetic resonance imaging (MRI)

To investigate the distribution of cells on the natural scaffold, we performed an MRI study of the acellular matrix seeded with amniotic fluid stem cells (AFSC) labeled with iron oxide particles.

Tubular intestinal scaffolds, with both ends tied up in a “sausage” fashion and an average length of 2 cm, were soaked overnight in sterile 1% penicillin/streptomycin (PS) in PBS. AFSC were seeded together with iron oxide particles (BioMag, 1.6 μm diameter, 1 mg/ml) and cultured for 24 h after which particle uptake was assessed. AFSC labeled with BioMag particles were seeded onto the scaffold at a concentration of 0.5 × 10^6^ cells/cm, in 2 mls Chang Medium supplemented with 10% fetal bovine serum (FBS) and 1% PS. Seeding was performed by means of pipetting directly into the scaffold lumen. The seeded scaffolds, placed in 6 well dishes, were maintained in a humidified atmosphere at 37 °C and 5% CO_2_ in an incubator. Cells were allowed to adhere to the scaffold for 2 h during which culture medium was gradually added every 30 min. Subsequently, the scaffold was cultured under stationary conditions in the incubator and the culture medium was refreshed every 24 h. To determine whether BioMag labeling could be used to check cell adherence differences due to cell death, after 24 h of seeding half of the seeded scaffolds were removed from culture medium and incubated in PBS/PS, to induce cell apoptosis. In addition, acellular intestinal matrices incubated with BioMag particles but with no cells were used as a negative control.

All the samples were processed for MRI analysis after 48 h of static seeding. Scaffolds with or without cells were tied off on one end with a 4-0 suture. The lumen was filled with 1% low melting point agarose (Fermentas Ltd., Hanover MD, USA) containing 8 mM Gadolinium-DTPA (Magnevist, Bayer AG, Berlin, Germany) and the other end was tied off too. After solidification, filled intestine samples were embedded using the same agarose gadolinium mixture as above in small glass vials (S Murray & Co Ltd, Surrey, England). Imaging was performed on a horizontal bore 9.4 T DirectDrive VNMRS system (Agilent Technologies, Palo Alto CA, USA) using a 26 mm quadrature birdcage volume coil (RAPID Biomedical GmbH, Würzburg Germany). For 3D imaging, a gradient-echo sequence with the following parameters was used: TE = 4.5 ms, TR 20 ms, flip angle 60°, 10 averages, field of view 26 × 13 × 13 mm, matrix size 1024 × 512 × 512 leading to an isotropic voxel size of 25 μm. Segmentations and 3D renderings were performed using Amira visualisation software (v5.2.2, Visage Imaging Inc., Andover MA, USA). The borders between agar and intestine lumen and agar and Serosa were segmented manually. Mesentery and hypo-intense regions indicative of high iron content were segmented using thresholds. In addition, to confirm the presence of live or apoptotic cells on the scaffold, samples were processed for H&E, Perls’ Prussian blue and cleaved Caspase 3. The number of cells in H&E sections of samples was counted in a blinded manner (assessor *n* = 5).

### 2.11 Chicken chorioallantoic membrane (CAM) angiogenic assay

To evaluate the angiogenic properties of the decellularized intestinal tissue *in vivo* we used the CAM assay as previously described [11]. Fertilized chicken eggs (Henry Stewart and Co., UK) were incubated at 37 °C and constant humidity. At 3 days of incubation an oval window of approximately 3 cm in diameter was cut into the shell with small dissecting scissors to reveal the embryo and CAM vessels. The window was sealed with tape and the eggs were returned to the incubator for a further 5 days. At day 8 of incubation, 1 mm diameter acellular intestinal matrices and polyester as a negative control, were placed on the CAM between branches of the blood vessels. Samples were examined daily until 6 days after placement wherein they were photographed *in ovo* with a stereomicroscope equipped with a Camera System (Leica) to quantify the blood vessels surrounding the matrices. The number of blood vessels less than 10 μm in diameter converging towards the placed tissues was counted blindly by assessors (scaffold *n* = 3, polyester *n* = 2), with the mean of the counts being considered.

## 3 Results

### 3.1 ECM permeability

Intestinal tissue was harvested from 25 adult Sprague–Dawley rats and treated with DET (Fig. 1A). Complete decellularization of the rat intestine was obtained after one cycle of DET. Following perfusion with the detergent-enzymatic solution both through the SMA and the intestinal lumen, the rat intestinal wall became macroscopically transparent with good preservation of the mesentery within 31 h (1 cycle of DET; Fig. 1B). Injection of 3 different dyes, Rosso Ponceau, Trypan blue and Rhodamine green in the vascular tree (Fig. 1C), venous tree (Fig. 1D) and intestinal lumen (Fig. 1E) respectively, showed preservation and patency of all three structures with no leakage following 1 cycle of DET. In comparison, further cycles of DET produced frail structures that were increasingly transparent with concomitant loss of the vascular integrity (data not shown).

### 3.2 Decellularization efficiency

**Fig. 2:**
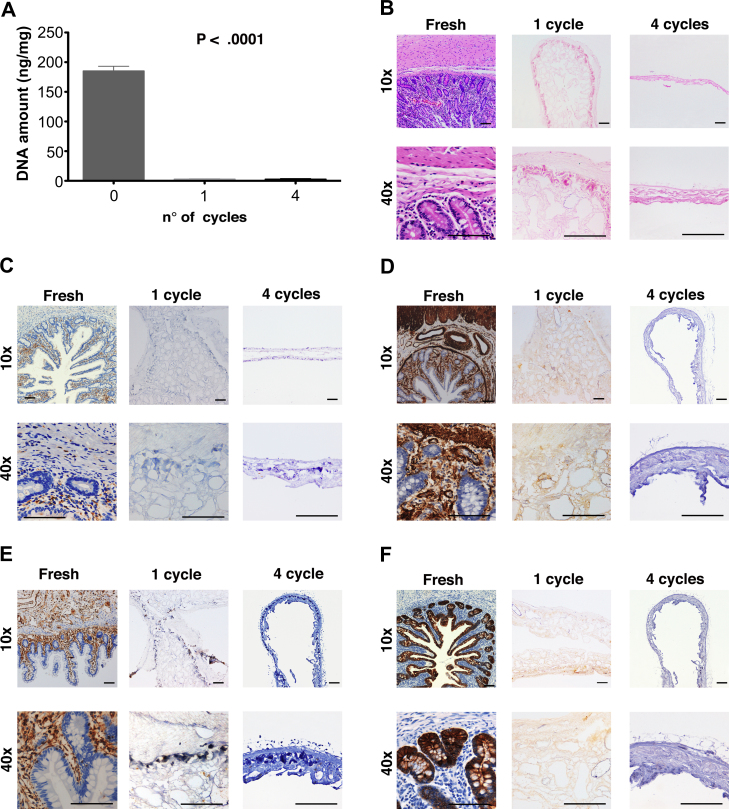
DNA quantification shows complete removal of DNA following 1 cycle of detergent-enzymatic treatment (*P* < 0.001) with no significant differences in DNA with additional cycles (A). H&E staining confirms the absence of nuclei and demonstrates preservation of structure following 1 cycle of treatment compared to fresh tissue and the loss of this upon prolonged decellularization as in cycle 4 (B). The lack of immunogenicity is confirmed with immunostaining for MHC-II (C). Immunostaining for SMA (D), Vimentin (E) and MNF 116 (F) confirms the absence of both mesoderm-derived and epithelial tissue markers with 1 cycle of treatment. The architecture of mucosa, submucosa and muscularis propria is lost with further cycles; H&E: hematoxylin and eosin, MHC-II: major histocompatibility complex II, SMA: smooth muscle actin, MNF 116: pan-cytokeratin.

DNA measurement showed that approximately 99% of DNA was removed by 1 cycle of the decellularization process when compared with fresh intestinal tissue (2.9 ± 0.3 ng/mg vs. 194.2 ± 12.0, *P* < 0.0001), with no significant difference between 1 and 4 cycles (Fig. 2A). Moreover, histological analysis with hematoxylin and eosin staining demonstrated absence of cell nuclei after 1 cycle of treatment whilst maintaining good architecture of the mucosa, submucosa and muscularis propria (Fig. 2B, 1 cycle). When treated with a higher number of cycles, the scaffolds lost their architecture and structural integrity (Fig. 2B, 4 cycles). Immunostaining with MHC-II was performed to determine whether cell surface markers were present on the scaffold, exhibiting a lack of immunogenic material following 1 cycle of treatment (Fig. 2C). To confirm the absence of both mesoderm-derived and epithelial tissue markers in the scaffold, the decellularized tissue was evaluated by immunostaining for smooth muscle actin (Fig. 2D), vimentin (Fig. 2E) and pan-cytokeratin (MNF 116; Fig. 2F). Staining was negative for all three markers after 1 cycle or more of DET, compared with strong staining of fresh native intestine.

### 3.3 Electron microscopy analysis

**Fig. 3:**
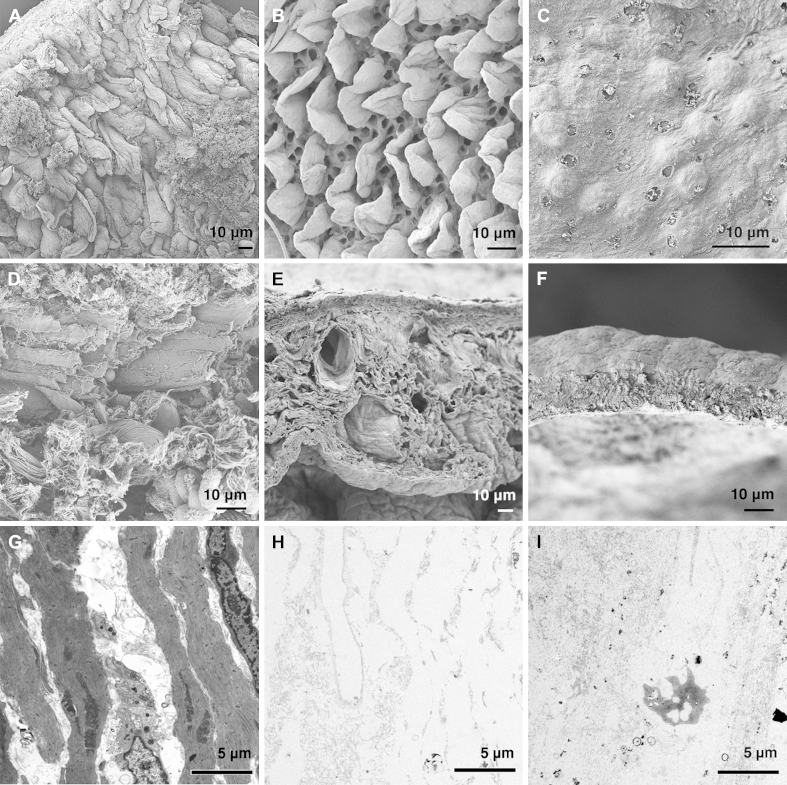
SEM and TEM of fresh intestine (A) and following 1 (B) and 4 (C) cycles of decellularization. While at 1 cycle the crypt/villus structure was completely preserved (B), this was completely lost at 4 cycles (C). SEM: scanning electron microscopy, TEM: transmission electron microscopy.

SEM of the intestinal acellular matrix showed preservation of the micro- and ultra- structural characteristics of the native tissue and confirmed the absence of cells. In particular, analysis of the luminal surface of the matrix after 1 cycle of DET revealed the presence of leaf-shaped villi and crypts at their bases (Fig. 3A–B). Interestingly, this aspect was conserved only for 1 treatment cycle while for further treatment cycles, the native villi structure was completely lost and there were no discernible signs of villus-crypt architecture even at higher magnifications (Fig. 3C). Wall section of the acellular matrix showed a collagen fiber network more tightly compacted in comparison to fresh samples (Fig. 3D and E). After 1 cycle, the microstructure of the intestinal wall and empty cavities that resembled vascular structures were clearly preserved (Fig. 3E). Further cycles of DET generated a further compact mesh of undistinguishable collagen fibers that resembled that previously reported for SIS [20] (Fig. 3F). Transmission electron microscopy confirmed the absence of cells in the decellularized tissue both after 1 and 4 cycles of DET (Fig. 3G–I).

### 3.4 Characterization of extracellular matrix components

**Fig. 4:**
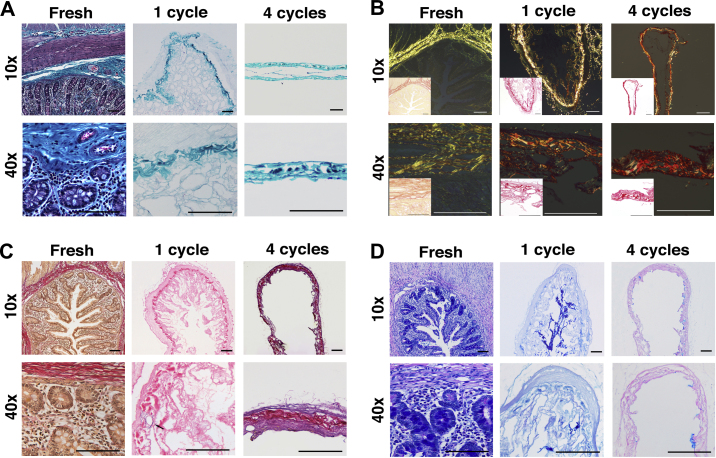
Characterization of the scaffold demonstrates preservation of structure, components of the ECM and removal of cellular elements. Masson’s Trichrome (A) and Picrosirius Red (B) staining confirm the maintenance of the connective tissue and collagen component of ECM. EVG staining (C) confirms the preservation of the elastin component around the blood vessels (black arrow) and Alcian Blue staining (D) the preservation of glycosaminoglycans; ECM: extracellular matrix, EVG: Elastic van Gieson.

**Fig. 5:**
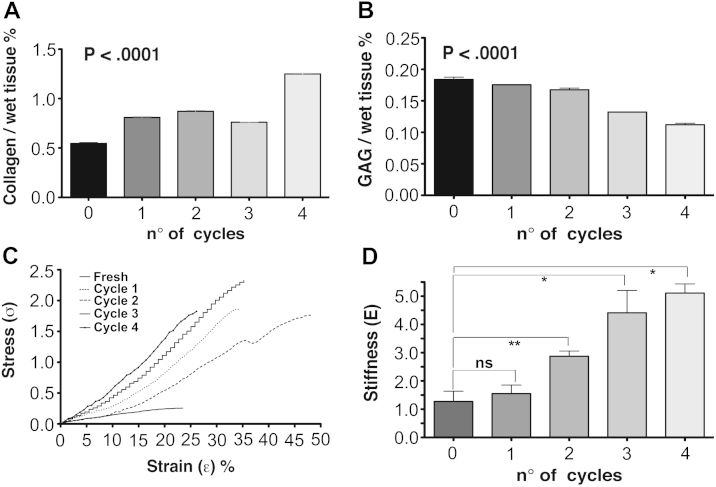
Collagen and GAG content of native tissue and decellularized samples at different cycles of DET (*n* ≥ 3 samples for all measures). Collagen content is increased in the acellular matrix (*P* < 0.0001; A) whilst GAG amount progressively decreases (*P* < 0.0001; B). Mechanical characterization of the acellular matrix: stress–strain curves show the tensile strength increasing with the number of cycles (C). No significant difference is observed in term of stiffness between native tissue and acellular matrix. (*n* ≥ 5 samples for all measures) (D); GAG: glycosaminoglycans.

In order to further characterize the structure of the scaffold and assess the effects of the detergent-enzymatic treatment to the ECM of the intestine, ECM components were evaluated. MT staining confirmed the almost complete removal of nuclear (purple) and cytoplasmic (pink) material after 1 cycle, together with preservation of connective tissue (blue) across the intestinal wall as well as maintenance of both transverse and longitudinal orientations of the muscularis propria (Fig. 4A). Similarly, collagen (Fig. 4B) and elastin (Fig. 4C) staining exhibited their preservation after 1 cycle of DET, whereas GAG were gradually depleted by the decellularization treatment (Fig. 4D). Additionally, in line with the histological findings, the quantitative assay showed that extracellular matrix collagen was preserved after the decellularization and its content (collagen/wet tissue weight) was significantly enhanced with an increasing number of DET cycles (*P* < 0.0001; Fig. 5A). In contrast, the amount of GAG declined progressively in the decellularized intestinal tissue as demonstrated by staining (*P* < 0.0001; Fig. 5B). Assessment of the mechanical properties of the natural acellular matrix showed a progressive rise in tensile strength with increasing decellularization cycles (Fig. 5C). After 1 cycle, samples (*n* = 5 for each cycle) were stiffer when compared with fresh tissue, but the differences were not significant (*P* > 0.05; Fig. 5D). However, sample stiffness increased with further treatment cycles (*P* < 0.018, 4th cycle vs. native) (Fig. 5D). Consequently, the maximum stress that the acellular matrix could withstand before rupture was higher compared to the native intestine.

### 3.5 Cell survival and angiogenic properties of decellularized intestine

**Fig. 6:**
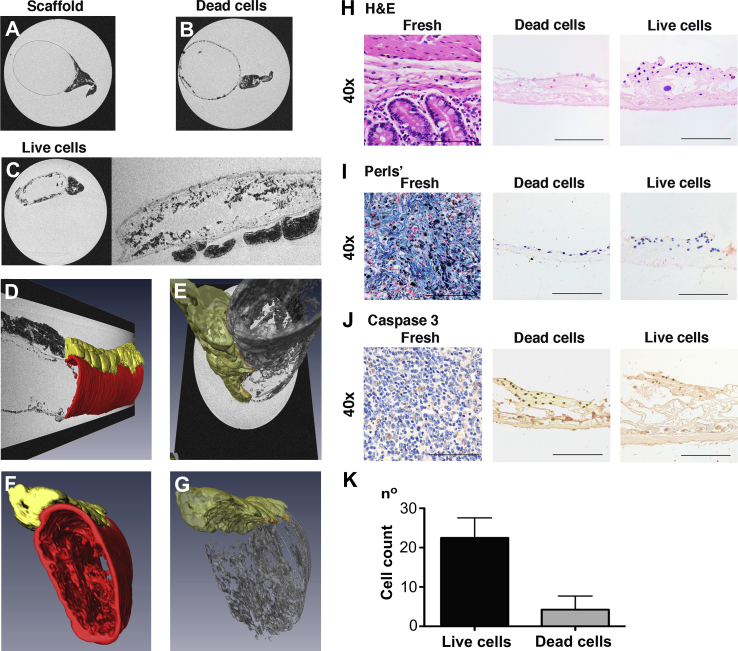
Magnetic resonance imaging as a viability test (A–K). Imaging of the acellular matrix shows its cylindrical structure with mesentery on the side (A). Matrix seeded with apoptotic AFSC shows partial repopulation and dissimilar distribution of the cells on the luminal surface (B). Scaffold seeded with live cells shows uniform repopulation of the lumen with cells distributed on the villi (both axial and longitudinal section) (C). Three-dimensional reconstruction demonstrates the matrix (red) overlying the cell layer (gray) and mesentery on the side (yellow) (D–G).H&E (H), Prussian blue (I), and Cleaved Caspase 3 (J) stains of scaffold seeded with both live and dead cells. Scale bars, 100 μm. Counting of cells seeded onto the matrix: number of live cells is significantly higher than dead cells (*P* = 0.01)(*n* = 5 for each measure) (K); AFSC: amniotic fluid stem cells, H&E: hematoxylin and eosin. (For interpretation of the references to color in this figure legend, the reader is referred to the web version of this article.)

MRI images of scaffolds following 1 cycle of decellularization showed a uniform cylindrical structure with the mesentery attached on the lateral side (Fig. 6A). When scaffolds were seeded statically with cells labeled with BioMag particles followed by induction of apoptosis and 48 h of incubation, there was partial repopulation, as shown by the hypo-intense regions in the lumen (Fig. 6B). The longitudinal section showed a dissimilar distribution of the cells as there was a higher amount in the inferior section compared to the superior section (data not shown). This is probably a sign of apoptotic cells detaching from the ECM. In the scaffold that had been seeded with BioMag-labeled live cells that had subsequently been allowed to attach and proliferate for 48 h, there was increased luminal hypo-intensity both in the axial and longitudinal sections with cells attached to the villous structures of the decellularized intestine (Fig. 6C). Three-dimensional reconstruction of segmented images showed even cell distributions (gray) within the scaffold (red) with the mesentery preserved on the side (yellow), confirming the *in vitro* biocompatibility of the decellularized tissue (Fig. 6D–G) (Supplementary Movie 1 online). H&E staining of the seeded scaffold demonstrated distribution of the live cells on the villi with strong cellular staining compared to the non-viable cell seeding (Fig. 6H). Perls’ staining was positive for the cells seeded on the scaffold, confirming their labeling with the BioMag particles (Fig. 6I) and Cleaved Caspase 3 staining confirmed the apoptotic nature of the non-viable cells (Fig. 6J). The scaffolds were counted for the cells present in the lumen and there were a significantly higher number of cells in the scaffolds seeded with live cells compared to those seeded with non-viable cells (*P* = 0.01) (Fig. 6K).

**Fig. 7:**
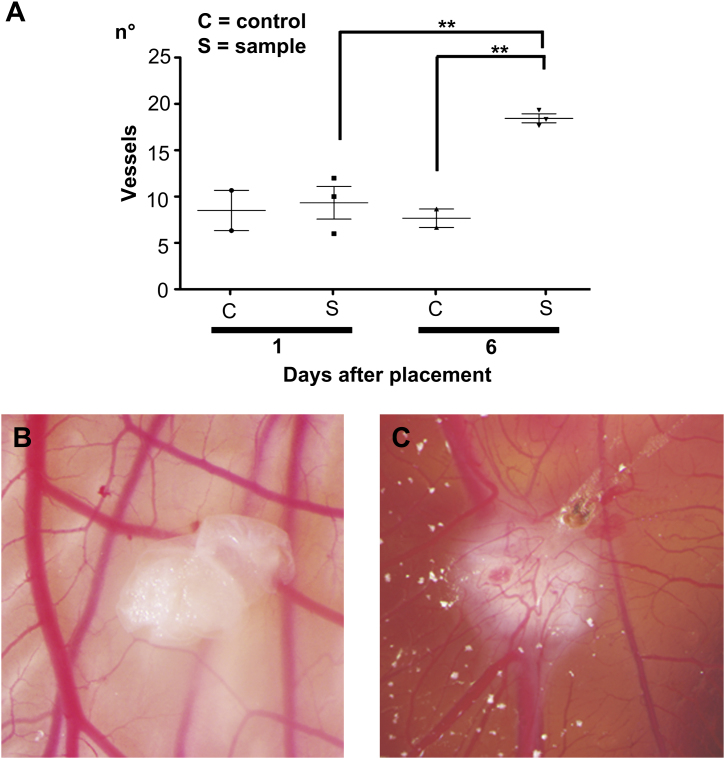
Pro-angiogenic properties of intestinal acellular matrix *in vivo* (A–C). Macroscopic quantification of converging vessels was blindly made for both intestinal decellularized samples and polyester membrane used as negative control (A). On day 6 after implantation, the number of vessels converging towards the intestinal matrices is significantly increased in comparison to the same samples at day 1 (*P* < 0.01) and to the polyester membrane that was used as a negative control (*P* < 0.05). Example of CAM at 1 day after implantation of intestinal acellular matrix: the sample of decellularized tissue is adherent to the CAM and starts to be surrounded by allantoic vessels (B). After 6 days of implantation, intestinal matrices are completely enveloped by the newly formed vessels, organized in a network (C).

To test the ability of the intestinal acellular matrix to attract blood vessels we used an established *in vivo* system [11] where the matrix was placed on the chicken chorioallantoic membrane (CAM). Samples of decellularized intestine and polyester membrane controls placed on the CAM were analyzed daily under a stereomicroscope. One day after placement on the CAM, intestinal matrices were adherent to the CAM and had started to be surrounded by allantoic vessels that grew towards the tissues. At day 6 after implantation, intestinal matrices were completely enveloped by the CAM and the vessels were organized in a network surrounding the tissue samples (Fig. 7B and C). To evaluate the pro-angiogenic effect of the intestinal acellular matrices on the CAM, vessel growth, (i.e. blood vessels converging towards the matrix) was quantified at day 1 and 6 in a blinded fashion. At day 1 after implantation no significant difference in the number of vessels growing towards the implanted tissues was observed. However, 6 days after implantation, the number of allantoic vessels converging towards the intestinal matrices was increased significantly compared to the same samples at day 1 (*P* < 0.01) and to the polyester membranes at the same time-point (*P* < 0.05; Fig. 7A).

## 4 Discussion

Recent advances in the field of regenerative medicine hold promise for the regeneration of different tissues and organs for clinical use. Tissue engineering of simple structures such as bladder [9], urethra [10] and trachea [12,13] has already been translated into patients. In that line, more complex modular organs such as the heart [22], lung [23] and liver [24,25] have been the subject of several studies in the last few years, while less extensive investigations have been applied to the intestine, probably due to the complexity of its structure and its functions.

In particular, the reproduction of the three-dimensional structure of the single intestinal functional unit (the crypt with the respective villus) still represents one of the major challenges for the development of functional intestine. The enormous surface area of the intestinal mucosa (approximately 300 m^2^ in an average person) is involved in the absorption of nutrients, secretion of hormones and enzymes and continuous epithelial regeneration. The latter represents probably the biggest challenge for the generation of functional intestine: intestinal epithelium is constantly renewed throughout life via a small population of stem cells that are able to completely renew the entire intestinal mucosa every 3–4 days, probably the fastest rate of turnover of any tissue in the body. It is believed that their disposition within the crypt helps the maintenance of the niche and the renewal of the entire epithelium. However, this structure is very difficult to mimic artificially and decellularized intestine described previously failed to preserve the original structure, which was lost together with the removal of the cellular component [31].

This is possibly related both to the detergents previously used and to the trauma received by the intestine during washing. In the present study we used continuous peristaltic delivery both via the intestinal lumen and vascular tree of a detergent-enzymatic solution to develop a natural acellular matrix after only 31 h. Characterization of this construct showed a complete removal of cellular elements with the preservation of the macro- and ultrastructural characteristics of the native tissue. Dye perfusion of both vascular tree and intestinal lumen showed the preservation of the three-dimensional vascular network (both arterial and venous) in the intestinal matrix after 1 cycle of detergent-enzymatic treatment. Histology and immunostaining analyses, together with quantitative assays and electron microscopy demonstrated the preservation of the main ECM component, collagen. Although GAG were significantly lost from the scaffold after several DET cycles, their depletion was not significant after a single cycle. Furthermore, mechanical tests showed that 1 cycle of detergent-enzymatic treatment does not significantly compromise mechanical properties of the intestine. The tensile modulus in the decellularized intestine did not change significantly, suggesting that the decellularization treatment does not affect the elastin component of the native tissue. However, the tensile strength of the decellularized samples was significantly higher compared to native tissue, which might reflect the relative increase of collagen in the acellular matrix and the reduction of other components (i.e. water) due to the absence of cellular elements. The preservation of the GAG and collagen is in line with the histological analyses.

We believe the natural ECM scaffolds produced using this method may represent an innovative platform for small bowel bioengineering since the ECM carries out secondary roles beyond mechanical support. These properties include the mediation of cell adhesion via integrin receptors [32], as well as positive influence on cell survival and proliferation by means of growth factors and cytokines [33]. In addition, preservation of the ECM of the villus-crypt unit may facilitate establishment of the regenerating unit. Furthermore, the ECM has been argued to influence differentiation via mechano-chemical transduction, as shown by the differentiation of MSC to neurons, muscle cells and osteoblasts when seeded on substrate mimicking the elasticity of each of those host tissues respectively [34]. This supports the need for tissue-specific scaffolds that can mediate signals to that effect and promote appropriate differentiation.

The benefits of the decellularization technique we used include the preservation of the ECM, which favorably influences cell fate, as well as of the macro- and micro-architecture. Our experience with intestinal decellularization has suggested that a combination of deionized water, sodium deoxycholate and DNase is superior to the use of Triton X-100 in preserving ECM structure whilst removing cellular material. The favorable angiogenic response in the CAM experiments argues for the preservation of growth factors and cell adhesion following seeding, confirming the concept of preservation of the small peptide sequences that binds to integrins. Sustainment of the macro-architecture is important in recreating the three-dimensional structure of the organ to be tissue-engineered as well as maintaining a vascular tree that will allow for cell and nutrient delivery and waste removal. The micro-architecture is of value towards maintaining the mechanical properties and porosity of the tissue that will be optimal for cell seeding and growth.

## 5 Conclusions

The present work shows that an acellular natural matrix can be obtained from rat intestine without disruption of structural and mechanical characteristics of the native tissue. The integrity of the vasculature could represent a valid support for the re-endothelialization and reconstruction of the vascular network. Moreover, the short time needed to obtain the natural scaffold could have a positive impact for its clinical application. The scarcity of tissue, which has thus far limited the application of allogeneic intestinal transplantation in the PN-dependent population could be overcome by the generation of functional engineered intestine. Moreover, the described method may be of interest to engineer intestine that could be used as a three-dimensional platform for drug testing and biological assays *in vitro* as an alternative approach to experimental animal models. Attempts to create a functional niche for cell proliferation and differentiation are ongoing in our laboratory.

## Figures and Tables

**Video S1:**
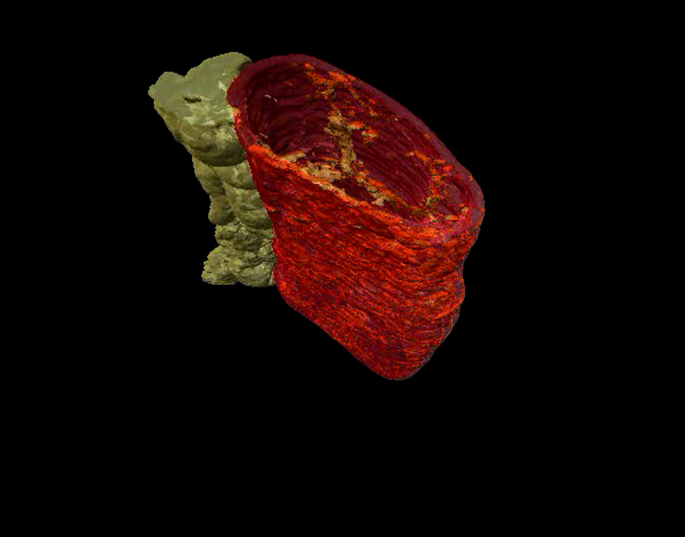
This movie shows high-resolution magnetic resonance (MRI) images of cells labeled with micron sized iron oxide particles seeded onto a natural small intestine scaffold. The movie starts transversing through short axis MRI images along the major axis of the intestine. A few seconds after the start, the volume rendering of segmented cells (brown, threshold of hypointensities), the intestine membrane (red) and the mesentery (yellow) is activated. (MPEG; 27 MB).
